# Aortic pseudoaneurysm caused by Tempofilter II inferior vena cava filter strut perforation: a case report

**DOI:** 10.3389/fsurg.2026.1895441

**Published:** 2026-08-11

**Authors:** Hao Zhang, Yue Wu, Zixin Tang, Michael Keese, Fei Wen, Feng Guo

**Affiliations:** 1 Thyroid and Galactophore and Vascular Surgery Department, Ba'nan Hospital Affiliated to Chongqing Medical University, Chongqing, China; 2 Otolaryngology Department, Ba'nan Hospital Affiliated to Chongqing Medical University, Chongqing, China; 3 Tongji Hospital, Tongji Medical College, Huazhong University of Science and Technology, Wuhan, China; 4 Department of Vascular Surgery, SLK Kliniken Heilbronn, Heilbronn, Germany

**Keywords:** abdominal aorta injury, deep vein thrombosis, inferior vena cava filter, inferior vena cava perforation, pseudoaneurysm

## Abstract

**Introduction:**

Inferior vena cava (IVC) filters are widely used in the management of pulmonary embolism (PE), although they carry risks of significant mechanical complications.

**Case presentation:**

We report the case of a patient with deep vein thrombosis and PE who developed an unexplained decrease in hemoglobin level following Tempofilter II filter placement and thrombolysis. Computed tomography angiography and digital subtraction angiography confirmed an abdominal aortic pseudoaneurysm caused by a penetrating filter strut. The filter was successfully retrieved, and the pseudoaneurysm was excluded via covered stent implantation, with favorable clinical outcomes.

**Discussion:**

Although rare, IVC filters can cause aortic pseudoaneurysm, a potentially life-threatening complication. Risk factors include advanced age, aortic tortuosity, and design-related propensity for migration of the Tempofilter II filter. This case study underscores the importance of thorough preoperative vascular assessment, prompt CT evaluation in patients with postprocedural back pain, individualized endovascular management, and patient education on activity restrictions.

**Conclusion:**

This case highlights the necessity of vigilant monitoring for rare but severe vascular complications following IVC filter placement.

## 1 Introduction

Inferior vena cava (IVC) filters are a well-established intervention for preventing pulmonary embolism (PE), a major cause of hospital mortality. Although filter-induced aortic injury is rare, it represents a severe procedural complication (1, 2). Herein, we report the case of an elderly patient who presented with acute left lower-limb pain, swelling, and PE, and subsequently underwent IVC filter placement followed by thrombolysis. Three days later, the patient developed back pain, a drop in hemoglobin levels, tachycardia, and hypotension secondary to IVC perforation with abdominal aortic pseudoaneurysm. Emergency covered stent repair and filter retrieval were successful. This case highlights the importance of standardized implantation practices, the early recognition of complications, and timely intervention to improve patient outcomes.

## 2 Case presentation

A 78-year-old woman presented with acute pain and swelling of the left lower limb that had persisted for 3 days, precipitated by prolonged sitting. Routine investigations revealed no evidence of thrombophilia, malignancy, or other provoking factors. Physical examination revealed pitting edema, mild erythema, and a slight increase in skin temperature of the left leg, without sensory or motor deficits. Limb circumferences measured at the ankle, 10 cm below the patella, and 15 cm above the patella were 23, 35, and 45 cm, respectively, on the left side, compared with 19, 31, and 38 cm on the right side (Supplementary Figure 1A). The left dorsalis pedis pulse was mildly diminished, whereas the right dorsalis pedis pulse was normal. Based on the aforementioned findings, there was no clinical evidence of phlegmasia cerulea dolens or acute limb ischemia. The patient had a 6-year history of hypertension but no history of diabetes mellitus, coronary heart disease, prior interventional procedures, smoking, or alcohol consumption. She also presented with chest tightness and cough, but no dyspnea or syncope. Doppler ultrasonography revealed extensive thrombosis involving the left iliac and femoropopliteal veins, while computed tomography angiography (CTA) demonstrated bilateral emboli in the main pulmonary arteries (Supplementary Figure 1B). These findings align with previous reports indicating that 70%–80% of PE cases originate from the lower extremity or pelvic veins, and that PE predominantly affects older adults (3).

**Figure 1:**
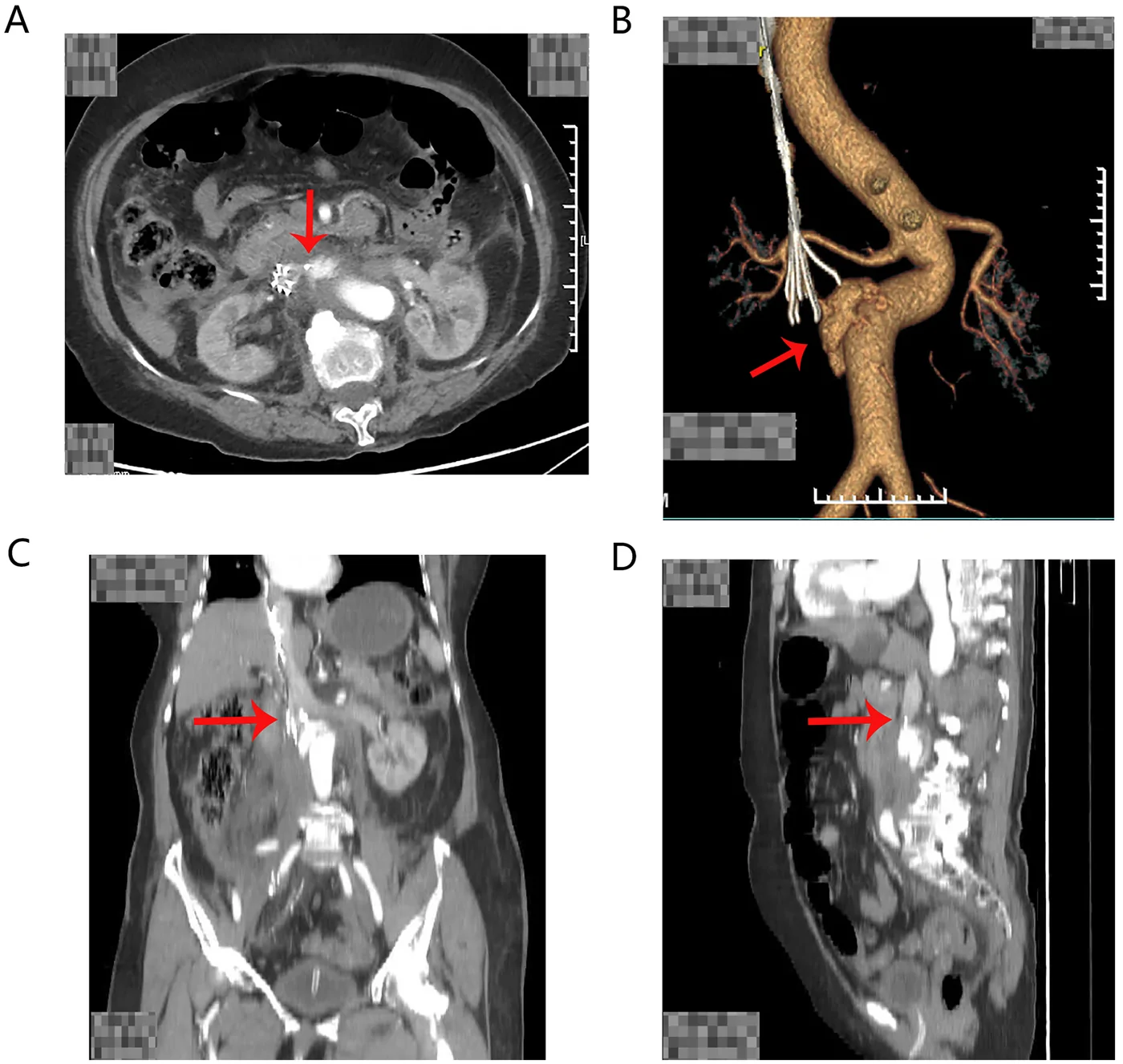
Abdominal aortic pseudoaneurysm: imaging findings. CT images in four planes show that a filter strut penetrated the IVC and entered the adjacent abdominal aorta, resulting in the formation of a pseudoaneurysm (red arrow). **(A)** Axial image. **(B)** 3D reconstruction. **(C)** Coronal image. **(D)** Sagittal image.

Based on laboratory findings and echocardiographic assessments, and in the absence of hypotension or other signs of hemodynamic instability, the PE was classified as low risk. Given the extensive iliofemoral thrombosis, the patient's and family's strong preference for active treatment, economic considerations, and current deep vein thrombosis (DVT) guidelines (4, 5), catheter-directed thrombolysis with temporary IVC filter protection was selected. Through right internal jugular vein access, IVC venography was performed to exclude filling defects and identify the renal vein orifices (Supplementary Figure 2). A temporary IVC filter (Tempofilter II, B. Braun, Germany) was placed 1 cm below the renal veins, and a 5F thrombolytic catheter (Unifuse, AngioDynamics, USA) was inserted into the fibular vein (Supplementary Figures 3A,B). Postoperatively, 100,000 U of urokinase was administered by continuous infusion every 6 h, combined with 5,000 IU of low-molecular-weight heparin administered subcutaneously every 12 h. On postoperative day 3, the patient developed back pain, a marked decrease in hemoglobin level (from 117 g/L on day 1 to 60 g/L on day 3; Supplementary Figure 3C), tachycardia (from 90 to 116 bpm), and hypotension (from 133/75 to 93/62 mmHg). Notably, blood transfusion had been performed after stent implantation. Anticoagulation and thrombolytic therapy were discontinued immediately. Based on the patient's clinical symptoms and imaging findings demonstrating filter morphology (Supplementary Figures 4A,B), filter perforation with abdominal aortic injury was strongly suspected. CT subsequently confirmed IVC perforation with an abdominal aortic pseudoaneurysm (Figure 1).

**Figure 2:**
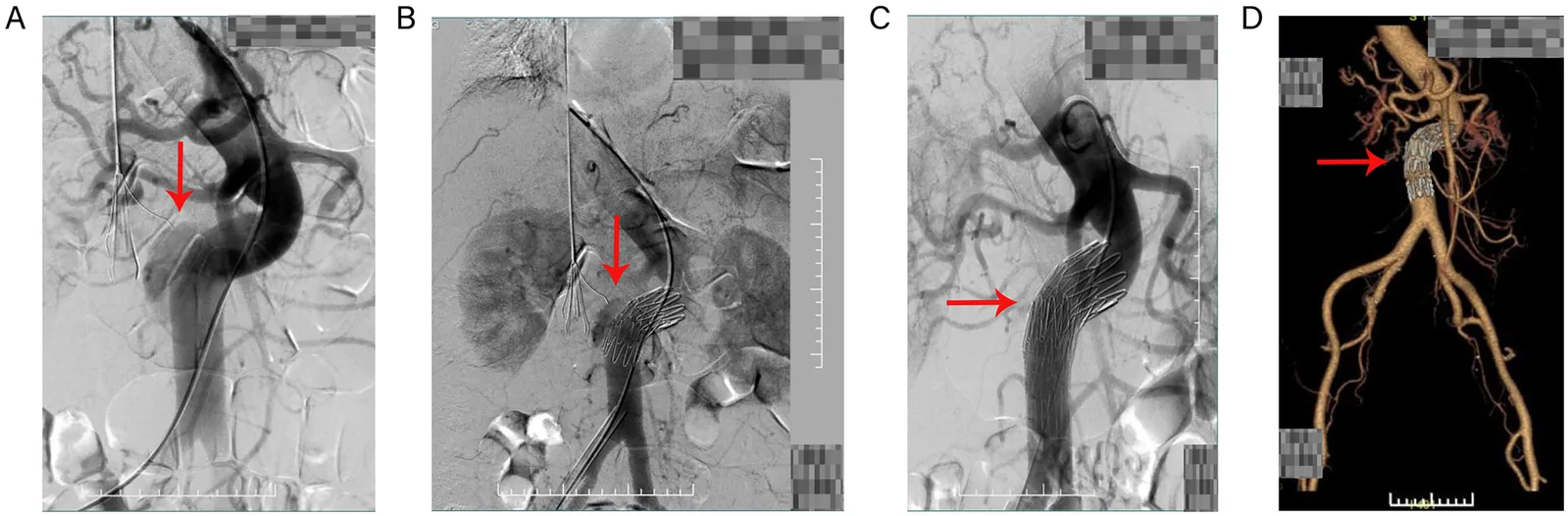
Endovascular management of the abdominal aortic pseudoaneurysm. **(A)** Angiography shows a filter strut adjacent to the abdominal aortic pseudoaneurysm (red arrow). **(B)** After deployment of the first covered stent, contrast extravasation persists, and the pseudoaneurysm remains visible (red arrow). **(C)** After deployment of a second covered stent, completion angiography confirms complete exclusion of the pseudoaneurysm without residual contrast extravasation (red arrow). **(D)** Postoperative follow-up confirms sustained pseudoaneurysm exclusion (red arrow).

Considering the recent anticoagulation and thrombolytic therapy, advanced age, and financial constraints, the patient underwent abdominal aortic covered stent implantation with concomitant IVC filter retrieval. The procedure was performed through a right femoral arterial approach using a 14F vascular sheath. Angiography showed marked migration of a filter strut with formation of an abdominal aortic pseudoaneurysm (Figure 2A). A covered stent (Cook, 58 mm × 24 mm) was then deployed; however, angiography demonstrated persistent contrast extravasation (Figure 2B). Consequently, a second covered stent (Cook, 58 mm × 24 mm) was placed distally as a bridging stent, with no residual contrast extravasation. The stents remained well clear of the superior mesenteric and renal arteries (Figure 2C). The filter was subsequently retrieved via a transjugular approach without confirmatory angiography; therefore, venous extravasation or arteriovenous fistula could not be excluded intraoperatively, representing a procedural limitation. After discharge, the patient received oral rivaroxaban 20 mg once daily for anticoagulation. Follow-up CT at 1 month demonstrated a well-positioned stent without contrast extravasation (Figure 2D). The absence of related clinical symptoms also suggested that no clinically significant IVC injury had occurred. The timeline of clinical events is detailed in Table 1.

**Table 1:** The timeline of clinical events.

Days	Clinical events
1	Laboratory tests (Hb 117 g/L); Tempofilter II placement with catheter-directed thrombolysis; postoperative urokinase 100,000 U q6h thrombolysis; low-molecular-weight heparin 5,000 U q12h.
2	Laboratory tests (Hb 112 g/L); continued anticoagulation and thrombolysis; gradual resolution of limb swelling.
3	Laboratory tests (Hb 60 g/L); patient complained of back pain; acute hemoglobin drop; CT revealed filter migration and perforation with abdominal aortic pseudoaneurysm; anticoagulation and thrombolysis discontinued; emergent endovascular repair with IVC filter retrieval and blood transfusion performed.
4	Hemoglobin stabilized; anticoagulation resumed.
5–14	Clinical condition stable; symptoms gradually resolved; no significant lower-limb swelling.
15	Discharged; prescribed long-term rivaroxaban 20 mg once daily.
30	Follow-up aortic CTA performed.

## 3 Discussion

In this patient with lower-limb DVT and PE, temporary IVC filter implantation followed by catheter-directed thrombolysis was complicated by back pain and a marked decrease in hemoglobin level. CT and angiography confirmed that a filter strut had perforated the IVC and penetrated the adjacent abdominal aorta, resulting in pseudoaneurysm formation. This case highlights the importance of recognizing aortic injury as a rare but severe complication of IVC filter placement.

IVC filter penetration is defined as extension of a filter strut >3 mm beyond the caval wall and may result in complications such as aortic pseudoaneurysm, arterial thrombosis, retroperitoneal hematoma, arteriovenous fistula, and aortic dissection. Abdominal pain is the most frequently reported symptom, occurring in 30.2% of cases (6). Although the mechanism of IVC filter penetration is not fully understood, proposed risk factors include filter geometry (conical filters with free struts), outward radial force, barb sharpness, strut stiffness, filter tilt >15°, small IVC diameter, and reduced caval wall strength (7). Conical filters have been associated with higher rates of fracture and penetration, while cylindrical or umbrella-like filters are associated with a higher incidence of IVC occlusion (8, 9). Additional contributing factors include abdominal trauma, cardiopulmonary motion, postural changes, and fluctuations in caval pressure (10, 11). The Tempofilter II is a tethered, barbless filter anchored to a subcutaneous cervical port via an indwelling jugular catheter. Physiological neck movements—including flexion, extension, and rotation—transmit traction through the catheter, allowing the filter to migrate up to 2 cm within the IVC. Accordingly, postoperative management includes 24 h of bed rest and restriction of vigorous neck movements, with flexion and extension limited to ≤30° during the first postoperative week to prevent excessive filter displacement. In this case, the Tempofilter II was selected primarily for economic reasons because the patient came from a rural area. The device can be retrieved at the bedside without specialized equipment (12), thereby reducing procedural costs and operator radiation exposure. However, these advantages are accompanied by tradeoffs, including filter migration related to cervical motion, increased nursing care burden, risk of catheter-related infection, and reduced patient comfort. From a technical perspective, the filter is easy to retrieve and aligns with current guidelines for the management of lower-extremity DVT. Nevertheless, the patient's advanced age and long-standing hypertension may have contributed to aortic sclerosis and reduced vascular compliance, resulting in aortic tortuosity and a reduced distance between the IVC and the abdominal aorta—anatomical features that may increase the risk of filter-related vascular injury. This case underscores the importance of considering preoperative aortic imaging, such as ultrasonography or CTA, in elderly patients to guide appropriate filter selection. It also emphasizes that prompt evaluation for filter-related injury in patients who develop symptoms such as back pain may facilitate timely diagnosis and definitive management.

Documentation of intra-abdominal aortic injury secondary to IVC filter is largely limited to case reports (13, 14). Kim et al. described a patient who underwent IVC filter removal, debridement, and reconstruction of the aortic and IVC. In another case, Jalili et al. performed open surgical repair with filter removal and reconstruction of the aorta using a Dacron patch. These reports emphasize that treatment strategies must be individualized according to clinical severity, institutional expertise, and patient comorbidities. In the present case, because of the patient's advanced age and hemorrhagic shock, an endovascular approach was selected for both filter retrieval and aortic repair. After considering the practicality and cost-effectiveness of abdominal endovascular devices, a CUFF stent was selected for abdominal aortic repair. Complete exclusion of the pseudoaneurysm was achieved using two CUFF stents. The endovascular approach effectively managed the complication while avoiding open surgery, and 1-month follow-up confirmed successful exclusion of the pseudoaneurysm.

However, several limitations should be acknowledged. First, preoperative aortic imaging was unavailable, precluding the assessment of preexisting aortic pathology (e.g., ulcer, dissection, or aneurysm), which could have contributed to underestimation of the lesion's complexity, including the possibility of multiple perforations and inadequate stent apposition in the tortuous aortic segment. Second, patient compliance and activity history (e.g., excessive neck movement, precipitating factors, or physiological motion related to diaphragmatic excursion or intestinal peristalsis) were not systematically documented, limiting the evaluation of the proposed mechanism of filter migration and vascular injury. Third, cavography was not performed after filter retrieval to exclude contrast extravasation from the IVC. Finally, long-term follow-up was not possible because the patient resided in a remote rural area with limited access to healthcare.

This case report, despite its limitations, provides practical clinical insights for clinicians and may inform future IVC filter design. In particular, it highlights the potential risk of vascular perforation associated with the device's permissible migration range, underscores the importance of the preoperative evaluation of perivascular anatomy in high-risk patients, supports prompt contrast-enhanced CT in patients who develop postprocedural symptoms such as back pain, and highlights the need for postoperative patient education and careful filter selection based on individual anatomical and clinical characteristics.

## 4 Conclusion

This case of Tempofilter II filter-induced aortic pseudoaneurysm suggests that the device's permissible migration range may contribute to the risk of vascular perforation, particularly in elderly patients with aortic tortuosity. It underscores the importance of the preoperative assessment of perivascular anatomy, prompt contrast-enhanced CT evaluation in patients who develop postprocedural back pain, meticulous patient education, and careful filter selection based on individual anatomical and clinical characteristics.

## Data Availability

The raw data supporting the conclusions of this article will be made available by the authors, without undue reservation.
