# Supplementary material for: Aortic pseudoaneurysm caused by Tempofilter II inferior vena cava filter strut perforation: a case report
Source: Front Surg. 2026 Aug 11;13:1895441. doi: 10.3389/fsurg.2026.1895441 (PMC13503547; doi:10.3389/fsurg.2026.1895441)
Supplement: Supplementary file 1 [file Supplementaryfile1.docx]

Supplementary Material

# 1 Supplementary Figures

## Figure 1. Clinical manifestations of the patient. (A) Marked swelling and increased circumference of the left lower limb (R: right, L: left). (B) Pulmonary artery CTA showing a thrombus in the main pulmonary artery (red arrow).

## Figure 2. Evaluation of IVC status. Angiography confirms the absence of filling defects in the IVC and identified the renal vein ostia.

## Figure 3. Thrombolytic treatment of deep veins in the lower limbs. (A) Angiography showing DVT. (B) Thrombolytic catheter placed via left peroneal vein puncture. (C) Trends in D-dimer (mg/L), fibrinogen (Fib, g/L), and hemoglobin (Hb, g/L) from postoperative days 1 to 3; D-dimer and Fib decrease, while Hb declines markedly on day 3. Values were not normalized and are shown only to demonstrate the 3‑day trend.

## Figure 4. Evaluation of IVC filter morphology. (A) After deployment, the filter is in a satisfactory position with adequate expansion. (B) Displacement of the filter anchoring leg toward the aorta is clearly observed (red arrow).
